# Facial recognition technology: regulations, rights and the rule of law

**DOI:** 10.3389/fdata.2024.1354659

**Published:** 2024-06-04

**Authors:** Mais Qandeel

**Affiliations:** School of Behavioural, Social and Legal Sciences, Örebro University, Örebro, Sweden

**Keywords:** unacceptable risk AI systems, facial recognition technology, responsibility to protect, duty to regulate, human rights, biometric data, the rule of law, legal certainty

## Abstract

Despite their pronounced potential, unacceptable risk AI systems, such as facial recognition, have been used as tools for, inter alia, digital surveillance, and policing. This usage raises concerns in relation to the protection of basic freedoms and liberties and upholding the rule of law. This article contributes to the legal discussion by investigating how the law must intervene, control, and regulate the use of unacceptable risk AI systems that concern biometric data from a human-rights and rule of law perspective. In doing so, the article first examines the collection of biometric data and the use of facial recognition technology. Second, it describes the nature of the obligation or duty of states to regulate in relation to new technologies. The article, lastly, assesses the legal implications resulting from the failure of states to regulate new technologies and investigates possible legal remedies. The article uses some relevant EU regulations as an illustrative example.

## 1 Introduction

The development and use of unacceptable risk AI systems, particularly facial recognition technology (FRT), are on the rise. As with benefits of every new technology, there are widespread concerns for and challenges to society and law. Legally speaking, these concerns arise in relation to human rights, discrimination, crime, the rule of law (RoL) and fundamental legal principles. These concerns are also accompanied with the question of responsibility, particularly in the absence of comprehensive legal frameworks to control and regulate the use and implications of these new technologies. There is currently an ongoing lively debate regarding whether FRT should be completely banned, limited or left unregulated. As of today, the development and use of new technologies are critically under-regulated, where the law has not yet been formed to deal with their manifested implications.

Facial recognition technology is a probabilistic technology designed to automatically recognize individuals based on their face in order to authenticate or identify them (European Data Protection Board, 2022). It makes it possible to compare digital facial images, collected through live video cameras (CCTV) or photos, to determine whether the compared images are of the same person. Comparing footage obtained from CCTV with images in databases is referred to as live facial recognition technology (LFR). When the videos are used with effect from data collected in the past, this is referred to as retrospective facial recognition (RFR). Additionally, mobile apps that allow photographing and checking individuals to identify them, this type is referred to as operator initiated facial recognition (OIFR).^1^ The technical aspects of how FRT operates will be discussed in section two of this article.

The design of these technologies and their deployment have increased concerns as they facilitate interference in a person's life, privacy and dignity. Unacceptable risk AI systems are those that are a threat to people and are categorized into (i) cognitive behavioral manipulation of people or specific vulnerable groups: for example voice-activated toys that encourage dangerous behavior in children, (ii) social scoring: classifying people based on behavior, socio-economic status or personal characteristics, (iii) biometric identification and categorization of people, and (iv) real-time and remote biometric identification systems, such as facial recognition.^2^ It is important to mention that facial recognition technology has been categorized as a unacceptable risk technology,^3^ as its AI system falls into the specific area of “biometric identification and categorization of natural persons', threats people and negatively affects fundamental rights.”^4^

States have the obligation to respect, protect and fulfill the human rights of individuals within their territory and territories under their effective control.^5^ This protection must safeguard against any abuses committed by state and non-state actors. The question of responsibility under international law is a matter of conduct. This means that states are generally not responsible for human rights abuses, unless such abuses can be attributed to them (Crawford, 2013b). Nevertheless, states must be held responsible if they fail to take appropriate measures to prevent, investigate and redress abuses.^6^ In other words, states are under an obligation to take a range of protective, preventive and remedial measures. States also have a duty to protect and promote the rule of law by ensuring equality before the law and fairness in its application, and by providing for accountability, legal certainty, and procedural and legal transparency. With the intensification of the use of new technologies, such as FRT, and their emerging ramifications, a state's duties and obligations require significant interaction to safeguard these legal principles.

Additionally, corporations that develop and provide sophisticated technologies, such as Google, Microsoft, Apple and Amazon, have full discretion to decide to whom they sell such products, either governmental agencies or private actors. These technologies are used for, inter alia, surveillance, censorship, and the interception of communications (Gates, 2011). Other tech companies, such as Facebook, Instagram, Clearview AI and Cambridge Analytica, collect data (images and videos) and globally sell it to state and non-state actors without the knowledge or consent of the individuals concerned (Rezende, 2020). Microsoft has, remarkably, asserted that FRT might be misused and regarded as invasive, called on governments to set up regulatory frameworks and introduced six principles to guide Microsoft's face recognition, namely, fairness, transparency, accountability, non-discrimination, notice, consent, and lawful surveillance (Sauer, 2018).

The deployment of unacceptable risk AI systems raises and magnifies concerns and questions in relation to the protection of basic rights and liberties as well as the general principles of law. These concerns have recently been addressed in the ample, available literature, where legal scholars have discussed the use of FRT in police investigations (Purshouse and Campbell, 2019; Fussey et al., 2021) and surveillance (Williams, 2020). They have also established that the use of FRT undermines the right to privacy (Lochner, 2013; Ringrose, 2019; Selinger and Hartzog, 2019; Wright, 2019; Barrett, 2020; Berle, 2020), the right to equality and non-discrimination (O'Neil, 2016; Noble, 2018; Benjamin, 2019; Human Rights Council, 2020), and the right to freedom of speech, leading to a spiral of silence (Stoycheff, 2016; Lynch, 2020).^7^ Additionally, empirical research has shown that FRT is bias against minority population, as it is less accurate for the faces of people between the ages of 18–30, particularly women and people of color (Klare et al., 2012). These findings are consistent with studies underpinning the argument that FRT disproportionately impacts minority groups and greatly impedes privacy (Buolamwini and Gebru, 2018; Grother et al., 2019; Shore, 2022). Within these concerns, empirical survey-based research has shown that individuals' acceptance to facial recognition varies given different political contexts and socio-demographic factors. The study found that “facial recognition technology enjoys generally highest acceptance among respondents in China, while acceptance is lowest in Germany, and the United Kingdom and the United States are in between (Kostka et al., 2021).” Only recently, some scholars have argued that this technology should be banned,^8^ while others have argued in favor of a calibrated trust-based approach to the use of facial recognition technology, which considers the relative risks and benefits (Chan, 2021).

The legal discussion must go beyond the implications of new technologies or whether they should be banned due to human rights violations. The legal thinking should also establish the connection with the rule of law, address how the law must intervene, control and regulate the use of new technologies and manage the question of responsibility. While discussions on regulating unacceptable risk and high risk AI systems have been ongoing, the perspective of the rule of law offers an important and unique contribution to the conversation. This article specifically focuses on the legal implications of a failure to regulate unacceptable risk AI systems, specifically facial recognition, in relation to the rule of law, which is absent in existing literature. Therefore, the approach, the emphasis on the rule of law perspective and the potential legal remedies resulting from the failure of states to regulate unacceptable risk AI systems provide for a novel contribution to the field.

This research does not intent to advance theoretical contribution or establish new legal theory. It uses the main premise of the theory of legal positivism for the purpose of providing a structured framework for analyzing and proposing regulation on unacceptable risk AI systems. This article does not intend to evaluate or assess relevant legal provisions or their efficiency; rather, it describes the need for a legal framework that systematically aligns with the principle of the rule of law and human-rights-based approach. Additionally, this article is not about debating the delineations of the applicable legal frameworks, but rather aims to showcase the potential contribution of law to control and assess implications of unacceptable risk AI systems. It brings examples of the approach of different jurisdictions concerning the regulations on FRT. The article directs its attention towards normativity of law, where “we analyse the concept in terms of reasons for action, that such reasons must be something more than prudential reasons, that the proper question is whether law gives us reasons for actions of the relevant type that we would not have without law (Bix, 2021).” In doing so, the article uses a variety of material, including laws and regulations, legal doctrines, case-law, and interdisciplinary literature review.

This article aims to contribute to the legal discussion by addressing how the law must intervene to control and regulate the use of unacceptable risk AI systems. It attempts to discuss the obligation of states to regulate these technologies, namely facial recognition, and their duty to protect through comprehensive regulatory frameworks in order to uphold the rule of law. The article starts with a brief introduction to FRT, an examination of biometric data collection and the use and implications of the technology. It then proceeds to describe the nature of the obligation of states to regulate unacceptable risk AI systems, arguing that states have an international obligation—it is thus not a matter of choice—to domestically regulate their use to ensure the respect of the rule of law. The article, lastly, assesses the legal implications resulting from the failure of states to regulate such technologies and investigates possible legal remedies. The article briefly brings some EU relevant regulations as an illustrative example, but not as an exhaustive case study. The article concludes that any regulations put in place must conclusively be in line with the rule of law principle.

## 2 Facial recognition and the use of biometric data

### 2.1 What is facial recognition technology and how does it work?

FRT relies on machine learning (ML), a form of artificial intelligence (AI), and is based on algorithms designed to collect and detect biometric information and features, through the automated extraction and digitisation of the spatial and geometric distribution of facial features (Berle, 2020, p. 9–10). ML and deep machine learning are techniques deployed to analyse complex variables or complexified real data (Tripathi, 2017).^9^ In technical terms, facial recognition algorithms can be referred to as “a process or set of rules to be followed in order to calculate or analyse facial characteristics … by a computer (Berle, 2020, p. 11).”

In its application of image processing and biometric systems, facial recognition can automatically classify and identify people's faces in any digital photograph or video feed (Berle, 2020, p. 2). The technology depends on the amount of data that is fed into it, the more the better. FRT is also connected to another closely related AI-technique called “affect (emotion) recognition technology,” which can be applied to the same data to infer personality traits, inner feelings and mental health (Wright, 2021). It analyses a person's “facial expression, voice intonation, gestures or movements and physiological aspects such as respiration, skin color, temperature, heartbeat, blood pressure, pupillary dilation (Faria and Almeid, 2013).” Affect recognition operates on “the premise that it is possible to automatically and systematically infer the emotional state of human beings from their facial expressions… [but] lacks a solid scientific basis.”^10^ Because very little is as yet known about affect recognition, this article primarily builds on the case of FRT.

Facial recognition system can be used to achieve one of the two goals: verification or identification. Verification is “(also known as 1:1 matching) is used to confirm that a person is who they say they are [… while identification is] (also known as 1:N or 1:many matching) is when software takes an unknown face and compares it to a large database of known faces to determine the unknown person's identity (Crumpler, 2020).” In addition to verification and identification, facial recognition can also technically serve to extract information about individuals' characteristics to analyse their faces and categorize their personal characteristics. FRT does not require human intervention to operate, which make it completely independent to identify or verify people only from images obtained or stored in the system on which it operates (Kortli et al., 2020).

Of the biometrics in use today, FRT is the least accurate, one of the most invasive and is rife with privacy concerns (Berle, 2020, p. 3). There are “two standard biometric measures to indicate the identifying power [these] are False Rejection Rate (FRR) and False Acceptance Rate (FAR) (Lin, 2000).” In other words, when individuals in videos and photos are subject to facial recognition algorithms, the results might be false in terms of rejection of acceptance, or might be accurate. Although facial recognition has improved its accuracy rate, accuracy is still considered an issue, where hundreds of people are wrongly flagged (European Union Agency for Fundamental Rights, 2019). Certain categories of people “may be more likely to be wrongly matched than others… [as there] are different ways to calculate and interpret error rates, so caution is required (European Union Agency for Fundamental Rights, 2019).” Because of its peculiarities, FRT has been categorized as one of the unacceptable risk AI systems that are considered a threat to people. For example, the European Union's AI Act, which will be briefly discussed later, bans real-time and remote biometric identification system, including facial recognition. Some exceptions, however, may be allowed for law enforcement purpose as “real-time remote biometric identification systems will be allowed in a limited number of serious cases, while post remote biometric identification systems, where identification occurs after a significant delay, will be allowed to prosecute serious crimes and only after court approval (European Parliament, 2023).”

### 2.2 Biometric data: mining and collection

It is important to properly define and critically analyse biometric data upon which FRT operates. According to the Oxford Dictionary, biometrics are “(1) a way of identifying people by their unique physical characteristics and (2) a person's unique physical characteristics.”^11^ The European General Data Protection Regulation (GDPR) defines biometric data as “personal data resulting from specific technical processing relating to the physical, physiological or behavioral characteristics of a natural person, which allow or confirm the unique identification of that natural person, such as facial images or dactyloscopic data.”^12^ Biometric data refers to the unique biological features that identify a natural person according to their intrinsic physical merits (Lynch, 2020). Fingerprints, faces, voice prints, palm prints, eye irises and DNA are all biometrics that exclusively identify a person's physical features. Differently put, biometric data is a form of digitalisation of the human body, which enables a view of human ethical and normative aspects (Van der Ploeg, 2005; Berle, 2020, p. 52).

This understanding is built on the premise that biometric data is: (i) personal data, (ii) a result of technical and technological processing, and (iii) directly connected to the physical, physiological and behavioral characteristics of a human being. These three grounds reflect a deconstruction of the legal concept of biometric data (Jasserand, 2016). Unlike other sensitive information, such as names or social security numbers, biometric data cannot be changed. Thus, once compromised, individuals have no alternatives and remain at extreme risk (Nguyen, 2018). Because of such inherent characteristics, biometric data must be protected.^13^ In addition, unacceptable risk AI systems, such as FRT, must be regulated at all levels in order to curb their implications for individuals and their biometric data. As biometric data is an integral part of the human body, it should naturally be treated as such, rather than as a product to serve the interests of state and non-state actors.

Faces are the most identifiable feature of a human being. The new regime of interoperability can disclose the identity of a person and their biometric features in no time at all (McClellan, 2020). Facial recognition is a type of biometric identification. Data collection and data mining are the basis upon which facial recognition operates. Typically, images and videos are uploaded on the internet by individuals, especially on social media platforms such as Facebook, Instagram, Twitter, LinkedIn, YouTube, TikTok, and Reddit. These images and videos are then stored by these platforms—tech companies—or harvested by other companies such as Clearview AI and Cambridge Analytica, later to be sold to state and non-state actors (Deibert, 2022). This is a convenient and cheap means of collecting data, it is the result of an active choice made by an individual to share their biometric and personal data. Nevertheless, the question of whether individuals are fully aware of the settings in which their biometric data is obtained, collected, retained, stored, saved, shared, sold and used by governments and companies remains debatable.

Collecting data forms part of the phenomenon of digital surveillance, which has relentlessly dominated the world and is employed by different actors through video cameras (CCTV) in public and private settings, drones, social media tracking, censored networks, the content of emails and internet searches (Human Rights Council, 2019b; Murray, 2019). The collection of data is also undergoing a constant process of real-time surveillance. The deployment of CCTV in streets, shops, shopping centers, at airports and security gates, on public transport… etc. is widespread around the world. The United Kingdom, the United States of America, France, Israel, China, and Argentina are only examples of countries which use CCTV excessively and which connect live-collected data with facial recognition technology.^14^ This deployment is a form of AI-enhanced mass surveillance and can take the form of verification/authentication by matching a live face to a photograph in an ID document, which is used in border controls (known as smart borders) (Del Rio et al., 2016). Other uses can take the form of identification by matching a photograph against a set database of photographs and detection by detecting faces in real time from sources such as CCTV footage and matching them to databases.

Moreover, every activity a person performs physically in real life or digitally on the internet is a form of data provision (metadata), mostly unwittingly and unwillingly. A well-known example of massive data collection is Edward Snowden's revelations to the world regarding the massive-scale surveillance and data collection of the US National Security Agency through the interception of private communications and access and storage of the data of individuals worldwide, including their biometric data (Amnesty International, 2022). Similarly, Europol, the EU's police agency, was also found to collect massive amounts of personal data and hack civilians' devices, including the interception of communication (Fotiadis et al., 2022).

### 2.3 The use and implications of facial recognition technology

Facial recognition technology is today used in many domains, especially law enforcement, crime prevention and security. It has above all been used by governments and businesses, and extensively in “border control, access control and secure login processes… [and possibly] to empower access to healthcare and banking (Berle, 2020, p. 10).” One of the current uses of FRT is at automated security gates, with the use of electronic passports and ID cards that contain personal information including digital photographs (Berle, 2020). FRT is also widely used by law enforcement agencies for the purposes of crime prevention, fighting terrorism, crime detection, borders, traffic regulation compliance, security access, and migration control (Berle, 2020, p. 19). The systems and databases of these agencies are largely fed with biometric data in order to identify suspects. FRT is further used in the domains of commerce, gambling and banking to enhance customer services and maximize benefits (Berle, 2020, p. 20–23). It can be used for simple functions such as unlocking smart devices as well as utilized for medical purposes, such as the diagnosis of Turner syndrome (Chen et al., 2018). Furthermore, it might be beneficial for the reunification of families, identification of victims of human trafficking and helping the visually-impaired to recognize faces. Affect recognition could, in addition, be used for psychological diagnoses and mental stress.

These benefits are undoubtedly creating an important societal advancement and objectives, whether for commercial or economic ends or for addressing public safety and law enforcement concerns. According to INTERPOL, the International Criminal Police Organization, for instance, “almost 1,500 terrorists, criminals, fugitives, persons of interest or missing persons have been identified since the launch of INTERPOL's facial recognition system at the end of 2016.”^15^ The INTERPOL Face Recognition System (IFRS) is claimed to contain images received from more than 179 countries. There is, however, no provided evidence or clarification on how and under what circumstances this system operates and the people subject to it. This might rise legal and ethical questions, although has led to public safety and law enforcement outcomes. Generally, many concerns have been discussed in relation to the application of FRT in these domains, especially in relation to privacy and consent.

Regardless of the potential benefits, FRT could be/has been misused by companies and public authorities alike. In fact, it has been used in disparate settings that lead to the misuse of data collected through photographs harvested from social media and live streams from mobile phones, web cameras and surveillance cameras on public transport, at airports and in stores for illegitimate and invasive purposes.^16^ Mass surveillance and the use of FRT by governments or oppressive and authoritarian regimes are very common. Such use has been justified for national security and public interest purposes. Specifically, FRT has been used in an extensive manner all over the world without any limitations, transparency, consent, scrutiny or accountability. It must be noted, however, that the use of FRT by law enforcement is an exception, i.e., the limited use for warranted purposes in the light of the principles of proportionality and necessity.

The European Parliament's Resolution on AI, especially FRT, is noteworthy, it provides that:

The different types of use of facial recognition… carry different implications for the protection of fundamental rights; … [their] deployment by law enforcement should be limited to clearly warranted purposes in full respect of the principles of proportionality and necessity and the applicable law; … the use of [FRT] must comply with the requirements of data minimization, data accuracy, storage limitation, data security and accountability… (European Parliament, 2021).

This assertion is of great significance to safeguard the principles, data accuracy and minimization considerations. Even if the technology becomes “accurate” and fulfills the requirements of accountability, lawfulness, fairness and transparency, there is a huge risk that the data that is fed into the algorithmic systems is fake. When it comes to accuracy and errors, “questions in relation to how easily a system can be tricked by, for example, fake face images (called “spoofing”) are important.”^17^ Deepfake technology is emerging and its use is intensifying, where the produced material could be—wrongly or purposefully—fed into the FRT system. This would, as a result, create an uncontrolled environment of FRT and lead to confusion and misconduct. The collection and mining of data could be an outcome of fabricated material, which corrupts data accuracy and accountability. This would ultimately lead to concerns regarding data security. Additionally, the acts of collection, retention and storage of biometric data increase vulnerability to cyberattacks and misuse by internal and external actors, creating vulnerable databases and requires an effective vulnerability mitigation model (Brandão, 2021). These acts will necessitate intensified work on data protection, information security, cyber security and accuracy. Today, cyberspace systems are targets in cyberattacks, particularly threats against confidentiality, integrity and availability of data (C.I.A Triad). The more we feed these systems with data, information and communications and store this data, the more vulnerable individuals, societies and governments will be to cyberattacks and potentially to corruption beyond restoration of entire systems.

New technologies have reshaped power and politics and contributed to the rise of digital repression (Feldstein, 2021). They have greatly facilitated massive and targeted surveillance, which have involved large-scale collection, retention, storage, analysis and usage of data and have become an integral part of state surveillance (Murry and Fussey, 2019). Corporations have similarly used these technologies to collect and store biometric data for profit, surveillance-based targeted advertising and political power-seeking purposes. Governments and corporations alike are the key players, whose actions should be scrutinized. In this context, this article subsequently finds a direct association between the responsibility of states to protect, including their duty to regulate state and non-state actors, and facial/affect technologies. This association is tightly coupled with the use of these technologies by all actors. This duty is discussed in the following section.

## 3 The responsibility of states to protect and the RoL: viewing FRT

Legal issues in relation to biometric data can be very intricate when deployed in new technologies. The traditional means is that these technologies and their implications are legally assessed to achieve data minimization, cybersecurity, accountability, and transparency for AI systems. The existing laws and regulations are focused on human rights issues and applied to protect individuals and prevent and remedy abuses. Hence, the growing legal and human rights uncertainties lead to the question of responsibility. The question pertains to who is responsible for ensuring that the use of these technologies is regulated, where states and non-state actors have an obligation not to infringe the rights of individuals. The nature of such obligations is directed at states through their regulatory powers. As such, there is a need to reassess the existing rules and establish new ones, if necessary. Such reassessment—which would supposedly lead to an up-to-date regulatory framework—is the obligation on states under international human rights law. This also entails that such an obligation includes protecting against the abuse of states, non-state actors and third parties and providing for a remedy to redress violations. This section examines the responsibility of states to protect and addresses the duty to regulate new technologies.

### 3.1 The responsibility of states to protect, respect and fulfill

It is internationally established that states have obligations and duties to respect, protect, and fulfill all human rights.^18^ These obligations require that states (i) may not interfere in the enjoyment of human rights or commit any abuses, (ii) must take every regulatory and necessary measure to protect everyone from the impairment of their rights by state and non-state actors, and (iii) must take affirmative action and appropriate measures to ensure that individuals fully enjoy their rights.^19^ The international consensus requires that human rights are protected in cyberspace and the real world alike (Rona and Aarons, 2016). The duty of states to protect, through their regulatory authority, is important to understand in relation to the use of FRT.

Sovereign states have the power to regulate for the public interest, which is embodied in customary international law. This regulatory power should address the concerns of society and the general interests of the general public and should afford compensation to individuals when damages occur. Laws and regulations are “conceived as that large subset of governance that is about steering the flow of events, as opposed to providing and distributing…. when regulators regulate, they often steer the providing and distributing that regulated actors supply (Braithwaite, 2011).” The state regulatory power—regardless of the form of sovereign commands—falls under a set of conditions where the law is fair, just and unarbitrary. This is on the assumption that the state respects human rights and upholds the rule of law. In this regard, provisions in international law direct their legal obligations to states. At the same time, non-state actors and individuals are reflected upon in certain provisions (Crawford, 2013a). States have positive and negative obligations. Positive obligations compel states to actively perform an action, while the negative obligations oblige states not to perform an action that obstructs the enjoyment of rights (Breakey, 2015). Protection by the law is a positive obligation, under which a state must develop “substantive and procedural guarantees to proactively protect [human] rights (Lavrysen, 2014).” It is also important to note that the protection of rights and liberties also includes a private actor acting on behalf of a state, for example, through a concession, public private partnership or as a collector of biometric data and selling the data to a state. This is, in addition to states being responsible for private actors under international law, to be considered as being attributed to the state itself and may be seen, legally speaking, as a further development of the state's obligation to protect human rights in all realms.

The obligations of states must also provide for an effective remedy for victims that enables individuals to “claim for a remedy before an independent and impartial body when a violation of a right has occurred or is likely to occur (Christian Courts, 2008).” This involves equality and fairness in the judicial and legal systems (Mckay, 2017). The Human Rights Committee (HRC) attaches the right to remedy with establishing appropriate tribunals and administrative procedures in order to address human rights issues under domestic law, including applying international instruments and constitutional provisions, conducting proper investigations, ensuring a cessation of perpetual violations, and entitlement to reparation.^20^ This obligation to regulate is generally derived from the positive duties of states under international law. In an explicatory attempt, the Limburg Principles on the Implementation of the International Covenant on Economic, Social and Cultural Rights, adopted in 1986, defined the scope and nature of state obligations and elaborated on the legislative measures that states must take in order to ensure full respect of the rights and freedoms of all.^21^ General Comment 3 of the UN Committee on Economic, Social and Cultural Rights (CESCR), in fact, recognizes that “legislation is highly desirable and in some cases may even be indispensable.”^22^ The enactment of a regulatory framework is not only a power at the hands of states but also becomes a duty of states to ensure that emerging challenges are legally addressed for the benefit of the public and the protection of freedoms, liberties and human rights. In the context of FRT, concerns in relation to cybersecurity and data protection should also be part of the whole picture.

The relevant main question here concerns whether the general duty of states to regulate applies to FRT. The implications of these technologies on human rights are apparent (Sauer, 2018; Purshouse and Campbell, 2019; Fussey et al., 2021) and cannot be ignored, and as a result, the issue of regulation has been widely discussed. The regulatory framework and use of facial recognition technology have been discussed internationally and regionally. At the UN level, the Human Rights Council has discussed the dangerous implications of AI on human rights. A UN group of legal experts and special rapporteurs have called for “an immediate moratorium on the sale, transfer and use of surveillance technology, until robust human rights safeguards are in place to regulate such practices (UN Human Rights Office of the High Commissioner, 2021).” The UN Special Rapporteur on freedom of opinion and expression has elaborated on the impact of new technologies on the promotion and protection of human rights in the context of assemblies, including peaceful protests, and, similarly, called for an immediate moratorium on the sale, transfer and use of surveillance technology, especially facial recognition, until human rights compliant regulatory frameworks are in place to ensure that state and non-state actors use these tools in a legitimate manner (Human Rights Council, 2019a).

It is clear and evident that there is a gap between law and practice, where the judiciary is faced with queries outside the legal framework. Of course, the legal system of each country and the power of judicial review plays an important role. Generally, this requires states to enact laws and regulations that address such effects before the use of these technologies becomes widespread and uncontainable. There is a distinction to be drawn between states and their interest in using their power to regulate. Some governments are gearing up their full efforts to protect their democratic values and liberties by regulating new technologies. While others benefit from the unregulated digital tools to tighten their control over those in opposition and dissidents.

In the context of such a distinction, it is important to consider the level at which a state exploits FRT and for what purposes such technologies are used. In situations where states are interested in ensuring that FRT is not misused by state and non-state actors alike, there is an increased possibility that comprehensive and preventive regulations are enacted. On the other hand, in situations where the state itself conducts massive operational digital surveillance, either for reasons of national security or oppression, it is very likely that these technologies are left unregulated or vaguely regulated. Issues pertaining from the use of FRT are coupled with legal concerns in relation to human rights, the rule of law as well as cybersecurity issues. The United Nations Special Rapporteur on the promotion and protection of the right to freedom of opinion and expression notes that contemporary digital technologies offer governments, corporations and criminals an unprecedented capacity to interfere with human rights (Human Rights Council, 2015). Regardless of whether it is a matter of a democratic or authoritarian/oppressive state, the unrestricted use of FRT is likely to undermine the rule of law and human rights. This use leads to the de-democratization of democracies and risks leading to oppression, creating incentives for more control.

The legal and societal implications might be much greater, and the use of these technologies more harmful in ways which cannot be foreseen at this stage. FRT could potentially be used in new areas and negatively disrupt traditional means in societies and legal systems. The potential expansion of FRT in other domains would in all probability produce other forms of harm and legal implications, which, unless legally unforeseeable, must be addressed by laws and regulations. Legal systems are already equipped with certain legal norms on which almost all regulations, laws and rules should be based. These new technologies that we are witnessing today challenge these legal norms, they do not obey them. Thus, the state duty to regulate new technologies is a significant tool to safeguard the legal order and to protect, respect and fulfill human rights.

### 3.2 The duty of states to regulate: principles of legality and legal certainty

The duty to regulate does not only derive from the international obligations to protect, respect and fulfill, it is also a necessity for a functional rule of law and legal order. The principles of legality and legal certainty are vital elements of the functional umbrella of the rule of law (UN High Commissioner for Human Rights, 2011). The rule of law functions to restrict the arbitrary exercise of sovereign power and is a principle of governance on which the legal order and systems—whether domestic, regional or international—have been built. Within the meaning of the rule of law principle, all persons and entities, including the State itself, “are accountable to laws… which are consistent with international human rights norms… [the rule of law] requires…to ensure adherence to the principles of … legal certainty, avoidance of arbitrariness, and procedural and legal transparency (UN Secretary-General, 2008).” The rule of law is a very general concept that includes the functionality of a democratic society, which respects human rights and accepts the superiority of the law. In the context of emerging technologies, this section establishes an understanding of how to ensure the “rule of the people by the people” in a setting where neither “rule,” “the people” nor “the State” remain as stable points in the transformative power of AI.

The principle of legality, in Latin: *nullum crimen sine lege*, means no crime without law, and provides that no conduct is banned or prohibited without the existence of a legal rule that bans such conduct (Varuhas, 2020). This, in the context of new technologies, means that, as a rule, the use of unacceptable risk AI systems is not restricted or criminalized until the law steps in to regulate and limit such use to prevent any adverse consequences on societies, freedoms and liberties, and legal principles. The industry of new technologies, especially AI and ML, is developing rapidly. The law, on the other hand, moves slowly and dilatorily, which makes legal rules unable to keep up with the emerging technologies, their use and legal implications. Thus, the law must have a degree of long-term vision in its application. The technology that poses an obvious risk and documented abuses require that the regulator weighs advantages against disadvantages. Nevertheless, in cases where technology can continuedly and unexpectedly be harmful, the proper approach is that regulators should address this harm with more restrictions to prevent violations.

The principle of legal certainty lies at the heart of understanding a state's duty to regulate. Legal certainty, as a general principle, necessitates that the law must be sufficiently clear, precise and accurate, while its effect must be predictable. It must “provide those [individuals] subject to legal norms with the means to regulate their own conduct and to protect against the arbitrary exercise of public power (Fenwick and Wrbka, 2016).” The European Court of Justice affirmed, in *Parliament v Council*, that the principle of legal certainty “requires that rules of law be clear and precise and predictable in their effect, so that interested parties can ascertain their position in situations and legal relationships governed by EU law.”^23^ The Court also specified, in *Intertanko Case*, that “[t]he general principle of legal certainty, which is a fundamental principle of community law, requires, in particular, that rules should be clear and precise, so that individuals may ascertain unequivocally what their rights and obligations are and may take steps accordingly.”^24^ Legal certainty has played an important role in the development of the legal order at all levels, whether international, regional or domestic. With these continuous technological and social changes, discussions on legal certainty suggest that the law should be more flexible and responsive (Fenwick and Wrbka, 2016, p. 2), which could grant the law a degree of leeway to expand the regulatory framework. Nevertheless, the principle of legal certainty remains a crucial element in the process of regulating new technologies, especially FRT and its use. Legal certainty is, in fact, a vital element of the rule of law that determines the liberties and freedoms of individuals and the range of state powers.

The principles of legality and legal certainty require that states regulate in order to achieve a level of stability within their legal order. Such stability can only be attained by enacting or amending laws and enhancing possibilities for judicial review. Judicial review offers interpretations on a case-by-case basis. However, these interpretations may differ if the legal provisions are unclear or non-existent. The need for appropriate national laws to curb the effects of new technologies, uphold the rule of law and protect human rights is vital. Appropriate national laws must have a clear purpose, comprehensive provisions, assurances regarding equality, legitimacy in objectives, an effectiveness in both application and remedy, and a proportionality in penalties. The overall understanding of the principles of legality and legal certainty relies on the clarity, effectiveness and comprehensiveness of the law.

States are also under the obligation to take steps to protect a range of rights from abuse, including in cyberspace. The state is required, for example, to take steps to protect people from abuses whether conducted by state personnel or third parties. It is important to note that the question of non-state actors and their connection to the state is of utmost importance. In the case of the participation of the state, attribution could be established. Are states responsible for the protection of rights and liberties when a private actor acts on behalf of a state, for example, through a concession, public private partnership, or as a collector of biometric data that sells the data to the state? Such a question is linked to the obligations of states, and whether the act performed by a state's contractors or private actors is attributable to the state. This stems from the state responsibility for private actors under international law. Additionally, the conduct of corporations and individuals requires civil and criminal liability. FRT is more complex than the exercise of rights on the internet, as it is a tool for human rights violations, oppression, undoing democracy and a threat to the well-established legal principles. Therefore, the failure of states to enact proper laws to protect human rights and promote the rule of law could lead to further legal implications and barriers to legal remedies.

Provisional work on regulating AI has already begun at the international level. In May 2019, the OECD principles on AI were adopted.^25^ They comprise five complementary values-based principles for innovative and trustworthy AI: (1.1) inclusive growth, sustainable development and wellbeing, (1.2) human-centered values and fairness, (1.3) transparency and explainability, (1.4) robust security and safety, and (1.5) accountability.^26^ According to these principles, the overall deployment of AI, which includes FRT as an AI-enhanced technology, should align with the principle of the rule of law, the respect of human rights and the respect of democratic values.^27^ The significance of the rule of law is the prominence of the determination to safeguard human rights as well as democratic values. Most prominently, the legal principles, which derive from the rule of law, namely: legal certainty, legality, and legal stability, are vital to provide fairness, social justice, and satisfactory legal remedies. In June 2019, the G20 supported the principles for stewardship of trustworthy AI (see footnote 28). The G20 was committed to supporting the respect of the rule of law, human rights and democratic values throughout the AI system lifecycle. These principles are of great importance when regulations on AI in general and FRT in particular are adopted. These international efforts could take the lead to establish an enforceable international legal framework or guidelines for domestic regulatory frameworks. In both cases, there must be effective and enforceable legal rules, beyond soft law or ethics.

At the national level, the UK serves as a very relevant example. The Court of Appeal in South Wales, in Ed Bridges v. South Wales Police, ruled that the use of automated facial recognition technology in a pilot project by the South Wales Police Force was not in accordance with the law for the purposes of ECHR art.8(2).^28^ This use does interfere with the rights to privacy, equality and non-discrimination. As there was no clear guidance on how and where the technology could be used and who could be put on a watchlist, a data protection impact assessment was inadequate and did not comply with the Data Protection Act 2018 Pt 3 s.64(3) (See footnote 29). Additionally, the Court found that the police had not taken reasonable steps to investigate whether the technology had a racial or gender bias, as required by the public sector equality duty; thus, the court declared that there are fundamental deficiencies in the legal framework in relation to the use of FRT (See footnote 29). In other cases: Metropolitan Police Service's trial of live facial recognition (2016–2019)^29^ and South Wales Police's trial of mobile phone facial recognition (2021–2022), studies have found that the deployment of FRT in these cases does not adhere to human rights, data protection or ethical requirements (Radiya-Dixit, 2022). However, there is no specific legal framework that regulates the permissive use of FRT in the UK by the Police. The only applicable rules are data protection, equality and human rights laws. It can be understood that the permissive approach to the use of FRT in the UK has been consistently leading to violations of human rights and data protection laws. Ostensibly, without a legal-restrictive approach, extensive and arbitrary use of FRT would only continue to exacerbate human rights and data protection breaches.

Efforts have also been made at the regional level. For example, the European Union has lengthily discussed the implications of AI on law and general legal principles. It is not the purpose of this article to discuss the European framework or provide for a comparative study. The purpose is rather to illustrate those efforts at the regional level, which may provide practical insights into the development of rules at the international level and to show how states' efforts to regulate the use of AI-enhanced technologies are of utmost importance. At the European level, by way of example, the GDPR, adopted in 2016, applies to AI for this very purpose and for limitations, which covers, *inter alia*, the prohibition of applying automated means on the data subject, the right not to be subjected to an automated decision, the protection of the fundamental right to data protection.^30^ More precisely, Article 9 of GDPR prohibits the processing of personal data that reveals origins or political opinions as well as processing of health, genetic and biometric data without explicit consent or necessity.

The European Court of Justice (ECJ), in Willems and others v Burgermeester van Nuth and others,^31^ adjudicated on whether collected biometric data falls within the protection of Regulation 2252/2004 art.4(3).^32^ The Court states that the Regulation did not require the Member States to guarantee, in their legislation, that biometric data collected and stored in accordance with that regulation will not be collected, processed and used for purposes other than the issue of the passport or travel document, since that was not a matter which fell within the scope of that regulation.^33^ On the contrary, the European Court of Human Rights (ECtHR), in Murray v. United Kingdom (1994), discussed concerns in relation to the retention of biometric data, related to the storage of photographs of convicted terrorists in Ireland. The Grand Chamber of the ECtHR held that the retention and storage of basic personal details about the arrested person, or even about other persons present at the time, is not outside the legitimate limits of the procedure for investigating terrorist offenses.^34^

The European Commission has also elaborated on whether AI-enhanced technology should be banned for privacy and data protection concerns (Heikkilä, 2021). The EU, including the EU Article-29-Data-Protection Working Party, incorporated these concerns in its European Data Protection Supervisor Strategy 2020–2024 and regularly discusses the various legal and regulatory issues related to AI. The discussion is, however, limited to how AI surveillance interferes in data protection and privacy. Recently, the European Parliament has called for a ban on FRT used in public places, and on predictive policing and a ban on private facial recognition databases (Heikkilä, 2021).

The fact that the European Union's effort to regulate AI can be used as a regional example is also demonstrated in other legislative frameworks. Followed by the European Commission's White Paper on Artificial Intelligence—A European Approach to Excellence and Trust of 2020 (European Commission, 2020), the Proposal for a Regulation of the European Parliament and of the Council (Artificial Intelligence Act) was published on 21 April 2021, suggesting harmonized rules on AI.^35^ While the European Council revised the version in November 2022,^36^ the European Parliament adopted final amendments to the Act on 14 June 2023 and it was approved on 8 December 2023. The Act on Artificial Intelligence includes the objective of ensuring that “AI systems… are safe and respect existing law on fundamental rights and Union values; ensure[ing] legal certainty…; [and enhancing] governance and effective enforcement of existing law on fundamental rights and safety requirements applicable to AI systems…”^37^ Importantly, the AI Act bans “real-time” and “remote” biometric identification systems, except for strict use by law enforcement, with the obligation of emotion recognition and biometric categorization disclosure and authorization (Veale and Borgesius, 2021).

Although the AI Act suffers from fragmentation and uncertainty,^38^ it serves as a first regional step—a first in the world—for a regulatory framework on AI, with the objective of protecting the values of fundamental rights, the rule of law, democracy, non-discrimination, data protection and human dignity. It provides a framework for a restrictive use of facial recognition as well as safeguards for the principles of legality and legal certainty. Nevertheless, more detailed protection of biometric data—beyond the issues of the internal market—could have been spelled out more clearly and with a more comprehensive legal treatment. Facial recognition, as other unacceptable risk AI systems, requires more than a general statement such as the “use of ‘real time' remote biometric identification systems in publicly accessible spaces for the purpose of law enforcement is also prohibited unless certain limited exceptions apply,”^39^ or “[it] should be subject to appropriate limits in time and space.”^40^ Even when transparency obligations^41^ serve as a basis for the use of a facial recognition system, consent is a more complex issue that should be dealt with in a regulation of this kind. Consent is used as a common legal basis for the collection of data, but not yet through the deployment of facial recognition technologies (Selinger and Hartzog, 2019). Even if implemented, it is difficult to fulfill the condition of consent in public or private places (Selinger and Hartzog, 2019). A full free will and complete awareness of consequences, conditions and usage as well as the ability to choose are aspects that are not likely to be fulfilled. This is because individuals would not have an alternative to consent. Most probably, when certain individuals explicitly refuse to be subjected to facial recognition, they will be perceived as suspects. The remaining question concerns the situations in which a state fails to enact proper laws to protect the rights and liberties of individuals in the age of facial recognition. The answer to this question is addressed in the following section in two main points of discussion: (1) legal implications and (2) accountability through legal remedies.

## 4 The failure of states to enact proper laws to protect the rights of individuals

### 4.1 Legal implications

The previous section examined whether states must ensure that FRT is regulated in order to achieve a level of respect for human rights and the rule of law. This responsibility comes jointly with a comprehensive legal framework to regulate new technologies that pose a high and great risk to society and law, including human rights. The failure of states to introduce such a framework could lead to dangerous legal implications beyond human rights violations. Such a failure might threaten the fabric of society and endanger the well-founded legal principles. The implications identified are (a) the normalization of the use of tools that contribute to the infringement of human rights and (b) instability in the legal order, creating uncertainty and legal vacuum. This section identifies such implications without discussing them in detail as it is impossible to do them justice within the limited scope of this article.

It is important to keep in mind that the biometric data that is collected is extremely sensitive and requires, under regulated situations, highly secure cyber systems. FRT can serve as an excellent tool of oppression at the disposal of governments (Hartzog and Selligner, 2018). Maximizing data collection and a massive extension of usage^42^ are worrying. From a holistic approach, the impact of data mining and surveillance would generate an unprecedented legal and societal dilemma. The absence of regulations to control FRT would allow for unrestrained data collection by either governments, companies or individuals. This, in turn, directs concerns toward the entities involved and means by which biometric data is obtained, collected, retained, stored, saved, shared and used.

Whether in a democratic society or under an oppressive regime, infringements of rights are very probable implications of the use of facial recognition. Surveillance and facial recognition actively embrace the destruction of privacy bringing increased and motivated security concerns (Kaya, 2006). Reaffirming the relevant legal literature (Sauer, 2018; Purshouse and Campbell, 2019; Fussey et al., 2021), the misuse of this technology leads to the infringement of privacy, equality and non-discrimination and basic freedoms, and undermines democracy and the rule of law. This discrimination is in all probability likely to occur on the grounds of ethnicity and gender. When used in public spaces, it is almost impossible for individuals to avoid being subject to facial recognition, creating public fear and insecurity. Such use of AI to maximize and amplify surveillance, especially with facial recognition, increases the potential for authoritarian control and oppression. In conflict areas, the use of this technology for surveillance is being intensified. This technology could be further used, justified for security reasons or military necessity, to suppress resistance, pose risks regarding adverse humanitarian consequences and heavily threaten human rights, particularly the right to self-determination. Elaborately, the impact of AI-enhanced surveillance puts democratic societies at risk, this risk becomes much more dangerous in less democratic societies and situations of conflicts.

The understanding of the rule of law includes the principles of legal certainty and legality. The rule of law is fundamentally undermined by the very existence and use of AI (Murry, 2021). When states fail to regulate facial recognition, as AI-enhanced tools, they enhance the capacity of these technologies to undermine the entire system of law and the rule of law. This is because at a fundamental level, the use of unregulated technologies to objectify human beings and study their biometric merits and features entails a change to human values, social structures and the integrity of the law. In the light of the rule of law, the failure of states to regulate causes uncertainty and instability in the legal order. Legal uncertainty simply occurs when individuals, within a state, are uncertain about the outcome or effect of the legal system in relation to others or their actions (Wagner, 2009). Legal uncertainty can be a result of either a non-existent statutory regulation or an unreliable basis for decisions (Wagner, 2009, p. 3). The non-existent statutory regulations or the absence of law, as a main cause of legal uncertainty, is the core dilemma that creates the negative legal implications of the failure of states to regulate. In the context of FRT, the absence of a regulatory framework that controls these unacceptable risk AI systems would firstly undermine democracies and the rule of law, secondly, allow for a chaotic collection, processing, and retention of data, and thirdly, lead to abuses of human rights.

There must be a proper and comprehensive law that aims at limiting the use of biometric identification systems including facial recognition by states and non-state actors. FRT that is considered as an unacceptable risk AI systems, would be prohibited, completely or partially, or need to comply with strict requirements. It is important that the users of such technologies are also restricted. The question of who is allowed to use FRT, governments, corporations, or individuals, should be addressed. In addition, there is a critical need to answer the questions of to what extent, for which reasons, in what places and with which limitations these actors are to be permitted to use these technologies and process biometric data. These questions must be clear in order to guarantee that the legal system and judiciary's functions align with the principle of the rule of law and human rights.

The implications of the failure of states to regulate is directly linked to the obstruction or prevention of the enforcement of legal rights and liberties. Such denial would lead to a complete fallout of the entire legal system and repudiate individuals to seek and access to justice. Denial of justice can be defined as “any gross miscarriage of justice by domestic courts resulting from the ill-functioning of the State's judicial system… [i]t may thus arise, broadly speaking, out of acts of the judiciary as well as of acts of the executive and the legislature affecting the administration of justice (Focarelli, 2020).” A state's failure to regulate is due to the malfunction of the executive and legislative powers in failing to address the emerging needs of a society. Such a malfunction leads to the inability of individuals to seek justice before judicial venues. It also deprives the judiciary from performing its role. It could be argued that there is nothing that prevents individuals from seeking justice under existing laws and claiming their rights. In the relevant context of the use of FRT technology, this argument does not hold as long as unacceptable risk AI systems are not regulated and remain an arbitrary tool in the hands of state and non-state actors. Ultimately, access to justice would not be granted, triggering state responsibility. It is established that the notion of the denial of justice remains, in fact, a very relevant concept for the determination of the international responsibility of states (Paulsson, 2005). The denial of justice can be the basis for an international claim (Paulsson, 2005). In other words, states may be held accountable for their failure to regulate, which leads to the obstruction of justice and infringement of human rights. This accountability or responsibility can be argued to be enforced in a form of legal remedies. Either under international law or domestic laws, the right to legal remedy might be the means to hold states accountable for a failure to regulate. This is the focus of the next section.

### 4.2 Accountability through legal remedies

States could be a threat to human rights. At the same time, states are the responsible guardians for respecting human rights within their borders and under their effective control. A failure to uphold such obligations, when not executed through regulating technologies that contribute to human rights abuses, triggers legal responsibility and remedies. These remedies are designed by domestic law and international law. Domestically, traditional laws, including constitutional norms, fall short of providing any succor for anonymity among the masses from the power of AI, simply because these laws have not foreseen such enormous powers and implications_._ In fact, even the current laws and doctrines are simply too antiquated to handle the implications and problems deriving from the use of facial recognition technology. The issue of a failure to regulate is, nonetheless, classical. At the international level, nothing is affirmatively clear. Yet, some international norms could be used to identify possible and available remedies and forms of reparation that would be included in cases where states fail, intentionally or unwittingly, to regulate and control the utilization of unacceptable risk technologies as intrusive tools.

In the absence of laws, only an analogy can be drawn to suggest legal remedies. FRT contributes to human rights violations, which leads to the question of reparation for human rights violations. States must regulate the use of FRT to halt or mitigate the negative effects of their use. The HRC, in its General Comment 31, notes that the enjoyment of human rights can be effectively assured by judicial administrative mechanisms to investigate allegations of violations.^43^ More importantly, the HRC requires a cessation of the perpetual violations and entitlement to reparation.^44^ There are two main points of discussion: (i) cessation of violations and (ii) reparation. The cessation of violations is an essential element of the right to an effective remedy. States must prevent violations and ensure that they cease to exist. Prevention is highly interconnected with a state's duty to regulate. In order to prevent and put an end to human rights violations, the tools that contribute to such violations must be regulated. This, as a result, suggests that facial recognition, as an unacceptable risk technology, is regulated to the extent that the issues of human rights, including privacy and discrimination, and data security and data collection are fully addressed. Reparation comes as a remedy through judicial and non-judicial avenues. The form or nature of reparations for human rights violations are made on a case-by-case basis. However, according to the UN Basic Principles and Guidelines, effective reparation has five forms, including: guarantees of non-repetition, restitution, compensation, rehabilitation, and satisfaction.^45^ The inclusion of proper protection, reparation, and non-repetition in future domestic legislation is indispensable to fulfilling the right to an effective remedy and the prevention of violations. This inclusion must address the peculiar legal implications of the use of FRT. The status of lawlessness and lack of remedies undermine the essence of the rule of law and the protection of human rights. Notably, putting comprehensive regulations in place is part of the state effort to prevent and remedy. When a state fails to fulfill its obligation to regulate emerging technologies that pose a high risk to the legal system and human rights, reparation is required. The need for regulation and the state's duty to regulate remain an urgency.

## 5 Conclusion

The rapid development and extensive adoption of facial recognition technology has brought about legal and societal challenges. The legal challenges and regulatory needs cannot be ignored. The main approach to tackle the need for a comprehensive framework is to put the emphasis on the duty of states to regulate facial recognition as an emerging unacceptable risk technology in order to prevent and remedy infringements of human rights and uphold the rule of law. This regulatory duty originates from the internationally established obligation where states have the responsibility to respect, protect, and fulfill all human rights. Additionally, a state's regulatory duty is vital to ensure that the principles of legality and legal certainty are achieved and provide for a level of stability within their legal order.

Potential legal and ethical implications and challenges of unacceptable risk AI systems, particularly facial recognition, might be difficult to predict. Future scenarios or trends in the regulation of unacceptable risk technologies would depend on the legal and ethical implications generated by the further development and use of such technologies. As AI systems continue to advance, there will likely be increased scrutiny and calls for regulations to address concerns around not only bias and privacy, but also around corporate and governmental accountability (developers and users). Balancing the benefits of AI systems with the risks they pose to society will be a significant challenge and need further scholarship attention. The widespread deployment of facial recognition technology would mostly circle around mass surveillance and its implications for civil liberties and democratic freedoms as well as its use in medical applications and diagnosis. Regulators may need to establish clear limitations on the use of facial recognition in public spaces, government surveillance programs, and commercial applications to prevent abuse and protect individuals and their rights.

A regulatory framework must offer comprehensive and legally binding rules that handle issues emerging from the use of FRT vis-à-vis the collection and processing of biometric data, ensuring the respect of human rights, upholding the rule of law, and delivering legal remedies. A complete legal prohibition is not necessarily an effective means to address the legal and societal implications of the use and misuse of FRT, although they are unacceptable risk technologies. It is important to consider that such technologies might be used by state and non-states actors alike for different and varying reasons. Perhaps, a complete ban could be part of the answer, nevertheless, it might lead to more violations, smuggling, secret use and illegal conduct.

Many questions must be resolved when determining the use of facial recognition, and these should include issues such as: (i) who is allowed to use these technologies, (ii) who is allowed to collect and process biometric data, (iii) should risk assessment and proportionality be tested, and (iv) how should the use of facial recognition comply with the requirements of data minimization, data accuracy, storage limitation, data security and accountability, as well as being lawful, fair and transparent, and following a specific, explicit and legitimate purpose? A clear legal framework can provide answers and solutions to these questions. Through the application of the law, potential gaps can be identified, where new legislation will become a necessity. Thus, a regulatory environment, including technological management, is needed (Brownsword, 2019). The legal framework of the duty of states to protect, their power to regulate the application of FRT and the potential legal implications need a comprehensive examination.

## Data availability statement

The original contributions presented in the study are included in the article/supplementary material, further inquiries can be directed to the author.

## Author contributions

MQ: Writing – original draft.

## Footnotes

^1^See: UK Government Home Office, *Police Use of Facial Recognition: Factsheet* (2023). Available online at: https://homeofficemedia.blog.gov.uk/2023/10/29/police-use-of-facial-recognition-factsheet/ (accessed 23 April 2024).

^2^See European Parliament. *EU AI ACT: First Regulation on Artificial Intelligence*. News (2023) Available online at: https://www.europarl.europa.eu/news/en/headlines/society/20230601STO93804/eu-ai-act-first-regulation-on-artificial-intelligence.

^3^See Report of the Special Rapporteur on the promotion and protection of the right to freedom of opinion and expression, Frank La Rue, UN Doc. No. A/HRC/23/40 (17 April 2013) paras 37-49.

^4^Proposal for a Regulation of the European Parliament and of the Council on laying down harmonized rules on Artificial Intelligence (Artificial Intelligence Act) and amending certain Union Legislative Acts (COM/2021/206 final).

^5^International Covenant on Civil and Political Rights (adopted 16 December 1966, entered into force 23 March 1976) 999 UNTS 171 (ICCPR) art 2.

^6^United Nations. *The Foundation of International Human Rights Law*. Available online at: https://www.un.org/en/about-us/udhr/foundation-of-international-human-rights-law (accessed June 17, 2022).

^7^The spiral of silence is “the significant chilling effect on an individual's willingness to publicly disclose political views when they believe their views differ from the majority.”

^8^Barrett. *Ban Facial Recognition Technologies for Children - And for Everyone Else*. 223.

^9^Deep machine learning is “based on a set of algorithms, which attempt to model higher level abstractions in data by using multiple processing layers with complex structures.”

^10^Human Rights Council, ‘The Right to Privacy in the Digital Age: Report of the United Nations High Commissioner for Human Rights' (13 September 2021) 48^th^ session UN Doc A/HRC/48/31, 7-8.

^11^Oxford Advanced Learner's Dictionary, Biometrics. Available online at: https://www.oxfordlearnersdictionaries.com/definition/english/biometrics (accessed October 25, 2023).

^12^The Regulation (EU) 2016/679 (General Data Protection Regulation), Article 4. (14).

^13^Nguyen, “The Standard for Biometric Data Protection.”

^14^See generally: Rodriguez, K. (2019). *Activists Worldwide Face Off Against Face Recognition: 2019 Year in Review*. Available online at: https://www.eff.org/deeplinks/2019/12/activists-worldwide-face-against-face-recognition-2019-year-review.

^15^INTERPOL, “Facial Recognition” Available online at: https://www.interpol.int/en/How-we-work/Forensics/Facial-Recognition (accessed April 29, 2024).

^16^David Leslie, *Understanding Bias in Facial Recognition Technologies: An Explainer* (The Alan Turing Institute 2020) 4.

^17^European Union Agency for Fundamental Rights, “Facial Recognition Technology: Fundamental Rights Considerations in the Context of Law Enforcement,” (2019), p. 9.

^18^UN Human Rights Committee, ‘General Comment No. 3: The Nature of the General Legal Obligations Imposed on States Parties to the Covenant' (2004) UN Doc CCPR/C/2 1/Rev.1/Add. 3 para 6-7.

^19^UN Human Rights Committee, General Comment No.3.

^20^UN Human Rights Committee, General Comment No. 31 (n 35) para 15.

^21^UN Commission on Human Rights, ‘Note Verbale' dated 5 December 1986 from the Permanent Mission of the Netherlands to the United Nations Office at Geneva addressed to the Centre for Human Rights (“Limburg Principles”), 8 January 1987, E/CN.4/1987/17.

^22^UN Human Rights Committee, ‘General Comment No. 31 (n 35) para 3.

^23^*Parliament v Council*, C-48/14, EU:C:2015:91 (12 February 2015) para 45.

^24^*Intertanko Case* C-308/06, EU:C:2008:312 (3 June 2008) para 69.

^25^OECD, “OECD AI Principles Overview” Available online at: https://oecd.ai/en/ai-principles (accessed November 16, 2023).

^26^OECD, “G20 AI Principles” (2019). Available online at: https://oecd.ai/en/wonk/documents/g20-ai-principles (accessed November 16, 2023).

^27^OECD, “G20 AI Principles,” Principle 1.2.

^28^*Ed Bridge v South Wales Police* [2020] The Court of Appeal C1/2019/2670, [2020] EWCA Civ 1058 (2020). Available online at: https://www.libertyhumanrights.org.uk/wp-content/uploads/2020/02/Bridges-Court-of-Appeal-judgment.pdf.

^29^See: Metropolitan Police, “Metropolitan Police Service Live Facial Recognition Trials” (August 2026–February 2029). Available online at: https://www.met.police.uk/SysSiteAssets/media/downloads/central/services/accessing-information/facial-recognition/met-evaluation-report.pdf (accessed April 23, 2024).

^30^General Data Protection Regulation (EU) 2016/679 of the European Parliament and of the Council of 27 April 2016 on the protection of natural persons with regard to the processing of personal data and on the free movement of such data, and repealing Directive 95/46/EC.

^31^*Willems and others v Burgermeester van Nuth and others* [2015] The European Court of Justice C-446/12 (16 April 2015).

^32^Council Regulation (EC) No 2252/2004 of 13 December 2004 on standards for security features and biometrics in passports and travel documents issued by Member States.

^33^Willems and others v Burgermeester van Nuth (n 46).

^34^*Murray v. The United Kingdom* [1994] 18731/91, Council of Europe: European Commission on Human Rights (27 August 1991).

^35^European Parliament and European Council, The Proposal for a Regulation of the European Parliament and of the Council (Artificial Intelligence Act) and Amending Certain Union Legislative Acts (COM/2021/206 final) 2021/0106 (COD) (2021). Available online at: https://eur-lex.europa.eu/legal-content/EN/TXT/?uri=CELEX%3A52021PC0206.

^36^Council of European Union, Proposal for a Regulation of the European Parliament and of the Council laying down harmonized rules on artificial intelligence (Artificial Intelligence Act) and amending certain Union legislative acts - General approach,2021/0106(COD), Brussels (2022). Available online at: https://artificialintelligenceact.eu/wp-content/uploads/2022/12/AIA-%E2%80%93-CZ-%E2%80%93-General-Approach-25-Nov-22.pdf.

^37^Artificial Intelligence Act, para 1.1.

^38^Veale and Zuiderveen Borgesius, “Demystifying the Draft EU Artificial Intelligence Act,” 110.

^39^Artificial Intelligence Act, para 5.2.2.

^40^Artificial Intelligence Act, para 8.

^41^Artificial Intelligence Act, para 5.2.4.

^42^Claude Castelluccia and Daniel Inria. *Impact Analysis of Facial Recognition Towards a Rigorous Methodology* (HAL 2020) 8–9.

^43^UN Human Rights Committee, ‘General Comment No. 3: The Nature of the General Legal Obligations Imposed on States Parties to the Covenant' (2004) UN Doc CCPR/C/2 1/Rev.1/Add. 3 para 15.

^44^UN Human Rights Committee, ‘General Comment No. 3.

^45^Basic Principles and Guidelines on the Right to a Remedy and Reparation for Victims of Gross Violations of International Human Rights Law and Serious Violations of International Humanitarian Law, Adopted and proclaimed by General Assembly resolution 60/147 of 16 December 2005.
